# Online Learning in Dental Education: Comparison of Perspectives of Lecturers and Undergraduate Students Between a Public and Private University: A Mixed-Method Study

**DOI:** 10.1155/2024/7389743

**Published:** 2024-10-16

**Authors:** Kwek Ching Yung, Liong Kah Lok, Budi Aslinie Md Sabri, Fawaz Pullishery, Sobia Bilal, Divya Gopinath

**Affiliations:** ^1^International Medical University, Kuala Lumpur, Malaysia; ^2^Faculty of Dentistry, Universiti Teknologi MARA, Sungai Buloh Campus, Sungai Buloh 47000, Selangor, Malaysia; ^3^Dentistry Program, Batterjee Medical College, Jeddah 21442, Saudi Arabia; ^4^Division of Prevention and Public Health Sciences, Pediatric Dentistry, College of Dentistry, University of Illinois Chicago, Chicago, IL, USA; ^5^Basic Medical and Dental Sciences Department, Ajman University, Ajman P.O. Box 346, UAE

**Keywords:** dental education, medical education, online learning, private, public

## Abstract

**Objective:** This study aimed to assess the undergraduates' and lecturers' perspectives on the implementation of online learning and to compare perspectives between private and public universities.

**Materials and methods:** The study followed a mixed-method design and involved dental undergraduates and lecturers from two study settings in Malaysia: International Medical University (IMU), a private university, and Universiti Teknologi MARA (UiTM), a public university. All undergraduates and lecturers were invited to complete an online questionnaire with evaluative statements regarding three domains: handling, didactic benefit, motivation, and an overall assessment. The comparison of perception between the private and public universities was analyzed using Mann–Whitney test. Focus group discussions (FGDs) were carried out for undergraduates and lecturers. Each FGD consisted of six people. The audio-recorded conversations were transcribed verbatim.

**Results:** Mean scores of perceptions regarding the domains, handling, and motivation were higher among undergraduates from public universities (*p*  < 0.05), while there were no differences for the didactic domain. There was no difference in perception of any of the three domains between private and public university lecturers. Mean scores for perceptions on the optimal amount of online learning in the future domains were higher among undergraduates from a public university (*p*  < 0.05), while lecturers' perceptions showed no difference. Thematic analysis of FGDs revealed that both groups from private and public universities felt that flexibility and a student-centered approach are the advantages of online learning. The inadequacy to cover the entire curriculum and lack of student engagement and interaction were highlighted as limitations.

**Conclusion:** Regardless of the university background, the undergraduates and lecturers were able to adjust to the online learning environment, which mainly reflected a positive perspective on the implementation of online learning. There were modest differences in the perceptions of dental undergraduates between private and public universities, while the perception of the lecturers did not show any difference.

## 1. Introduction

“Online learning” refers to education that occurs over the internet synchronously and asynchronously. Online learning can also be considered as one sort of “distance learning,” which refers to any learning that takes place over a long distance rather than in a typical classroom [1]. Since 1990, online learning has been an adjunct teaching tool for distance education [2]. Technology has transformed the teaching and learning process, and today, online learning can be conducted anytime from any location [3–5]. According to the Sloan Consortium, online enrollments are growing faster than the broader student population, and higher education institutions expect this trend to continue [6].

Lecturers/tutorials, simulation training, and clinical skills training are the three main components of dental education [7]. These three components, particularly in the case of simulation training and clinical skills training, necessitate intimate, close human interactions [8–10]. However, during the recent pandemic, all universities, including medical and dental universities, had to undergo an abrupt and extensive shift from traditional learning to a solely online learning environment, which was challenging for students and academics.

Online learning is a more flexible and effective way of teaching and learning since it facilitates learning with easy administration and accessibility while consuming fewer resources and time. Students can effortlessly access the study content regardless of time constraints. Students also learned to be self-directed learners, which is a critical skill for encouraging health professionals to continue studying throughout their careers [11, 12]. However, there are a lot of disadvantages as well. The lack of immediate instructor feedback and response is essential for online learning [13]. Although online learning allows students to continue learning off campus, it is a highly technology-dependent learning mode [14, 15]. Laptops for both the teacher and the student, an internet system, and the knowledge and ability to handle such digital devices are crucial factors. These characteristics are influenced by the country's socioeconomic situation, the dentistry school's budget, and other instrinsic factors including instructor and student inherent motivation [16–18].

The recent pandemic has undoubtedly intensified the emphasis on online learning in education. However, we expect this transition to become a lasting trend in dental education as several studies have acknowledged the benefits and value of online learning in determining success [19]. Nonetheless, the majority of studies focused entirely on the perspectives of students [20–23]. Only a few studies have looked into implementing online learning in dentistry education from both the students' and lecturers' viewpoints [10, 18, 24]. This mixed-method study will help to explore the perspectives of undergraduates and lecturers further, using a qualitative and quantitative paradigm to gain a more holistic and thorough insight. Thus, this study aims to evaluate the undergraduates' and lecturers' perspectives on online learning and compare the perspectives between private and public dental universities. This will help us identify the gaps and provide recommendations for the efficient development of online courses by triangulating qualitative and quantitative findings.

## 2. Materials and Methods

### 2.1. Conceptual Framework and Research Question

After reviewing the literature, we developed a conceptual framework with individual perceptions categorized into three main domains that may impact the effectiveness of online lecturers' and learners' teaching and learning by students. Based on this, we hypothesized that there would be no difference in undergraduates' and lecturers' perceptions of online learning at private and public dental universities during the challenging time of the SARS-CoV-2 pandemic. So, the research question for this research is as follows: “Are there any differences in the undergraduates' and lecturers' perceptions towards online learning during the challenging time of the SARS-CoV-2 pandemic between private and public dental universities?”

### 2.2. Sampling Procedures and Participants

This study was conducted in Kuala Lumpur, Malaysia, and included one private university, International Medical University (IMU), and one public university, Universiti Teknologi MARA (UiTM). The study sample consisted of all dentistry undergraduates and lecturers from the two study settings. Stratified convenience sampling was used, and the target sample size for dental students was 217, determined by identifying the smallest acceptable demographic subgroup comprising dental students from both universities with a population size of 500, with a ±5% margin of error, and with a confidence level of 95%. The lecturers' sample size was 44 with a ±5% margin of error and a confidence level of 95%. Students from semesters five, six, and seven were actively encouraged to complete the online questionnaire, although participation was voluntary. The study participants' names and other personal information were kept private and confidential. The study was explained to the participants, who were asked to sign a consent form. The following study has been approved by the IMU Joint Committee on Research and Ethics (Project ID: BDS I-01/2021(26).

### 2.3. Quantitative Phase

#### 2.3.1. Online Questionnaire

All the undergraduates and lecturers associated in the study settings were asked to complete an online questionnaire that included evaluative statements regarding three main domains: handling (how students or lecturers deal with online learning), didactic benefit (how students or lecturers perceive online learning as beneficial to dental education), and motivation (how students or lecturers were enthusiastic for online learning), along with a final overall assessment. Furthermore, the questionnaire for lecturers contained additional aspects regarding knowledge gained in providing online education. They were to select within a range from strongly agree to strongly disagree using a five-point Likert scale. Besides that, students and lecturers were asked how much online learning they would like to have in the future curriculum (independent of COVID-19). Finally, questions about demographics were posed. The respective questionnaires were pilot-tested on 20 random students and five lecturers and adjusted appropriately.

#### 2.3.2. Statistical Analysis

Collected data were entered and analyzed using IBM SPSS 23.0 software. The internal consistency reliability of the questionnaire was measured using Cronbach's alpha. Descriptive statistics, including means with 95% confidence intervals and standard deviations, were calculated. Depending on the normality of the data, a comparison of the difference in variables between private and public universities was analyzed using Mann–Whitney test. *p*  < 0.05 was considered statistically significant.

### 2.4. Qualitative Phase

#### 2.4.1. Focus Group Discussion (FGD)

FGDs were held for undergraduates and lecturers for private and public dental universities, respectively. A total of four sessions of FGDs were held separately for undergraduates from a private university, undergraduates from a public university, lecturers from a private university, and lecturers from a public university. Each focus group consisted of six participants. A random sampling technique was employed, and volunteers were selected among different cohorts for undergraduate FGDs, while lecturers from various specialties were chosen for lecturers' FGDs.

#### 2.4.2. Respondent Engagement

Two researchers facilitated the FGD based on the semistructured study guide containing grand tour and micro tour questions. The focus group was held only in English using Microsoft Teams software. Participants were given the information sheet and asked to sign consent before data collection. Each FGD was audio-recorded and lasted 30–45 min.

#### 2.4.3. Data Analysis

All audio recordings were transcribed verbatim using Microsoft Teams. Individual written consent was obtained. After data analysis, all audio-recorded conversations were discarded to maintain participant confidentiality.

## 3. Results

### 3.1. Quantitative Phase

#### 3.1.1. General Information

Two hundred twenty-seven dental undergraduates (IMU:89 UiTM:138) and 33 lecturers (IMU:17 UiTM:16) completed the questionnaire. Cronbach's alphas of the undergraduates' and lecturers' questionnaires were 0.786 and 0.678, respectively. There was a significant correlation between the three domains: handling, didactic benefits, and motivation.

#### 3.1.2. Handling Domain

Overall, undergraduates assessed the handling domain positively. They agreed that the software requirements for online learning were economically achievable, and the tech checks prepared them sufficiently for online learning. Further, most undergraduates acknowledged that online learning was well-structured and that the level of aspiration was high, indicating that they could follow the teaching content and did not feel over- or under-challenged (Table 1). The lecturers shared similar perspectives as the undergraduates regarding the handling domain (Table 2).

#### 3.1.3. Didactic Benefit Domain

The majority of undergraduates preferred face-to-face learning over online learning. However, they acknowledged that online learning is a good alternative for theoretical education but not for practical education because it makes them feel less prepared for the curriculum. They have difficulty adapting to online learning because it is more distracting and less interactive than face-to-face learning. They prefer online learning on the basis of saving time, the lectures being recorded, and the immediate access to online search in case of any doubts (Table 1).

Almost all lecturers believed online learning was adequate to replace face-to-face learning during the pandemic. They discovered that delivering knowledge and techniques via online education is more complex than educating in person. Furthermore, the students' attendance, interaction, response rate, and discipline were significantly lower. Nevertheless, most felt online learning saves time and allows for more extensive explanations because they can share their screen and search for compatible images (Table 2).

#### 3.1.4. Motivation Domain

More than half of the undergraduates agreed that using online platforms motivates them to learn (Table 1). Online platforms encouraged the lecturers and provided additional value as they could use it for other training purposes (Table 2).

#### 3.1.5. Comparison Between Private and Public Universities

Significant differences could be noted in the perceptions of dental undergraduates between private and public universities toward the handling and motivation domains of online learning in the challenging time of the COVID-19 pandemic between private and public universities (*p*  < 0.05), with public university students showing a more positive perception. However, there was no difference in the perceptions toward the didactic benefit domains (Table 3). On the other hand, there was no significant difference in the lecturers' perception toward all three domains of online learning between private and public universities (Table 4).

#### 3.1.6. Overall Assessment

For the students' group, there were some similarities in the perceptions of dental undergraduates of private and public dental universities. Compared to face-to-face learning, more than 50% believed online learning is more modern and time-saving. Despite this, they felt that face-to-face learning is also crucial since it is more fun and interactive, has fewer technical issues, and allows them to focus better during lectures. Meanwhile, dental undergraduates at public universities agreed that online learning would provide them with better knowledge transfer (54.3%), easier participation (39.1%), and more tips from lecturers (56.5%). In contrast, dental undergraduates at private universities believed that it is almost equivalent to face-to-face learning. Almost half of the dental undergraduates from private university felt that online learning is less tense and stressful. In comparison, 44.2% of the public dental undergraduates thought there was no difference between both modes. Regarding efficiency, private dental undergraduates were more inclined toward online learning (43.8%), while public dental undergraduates preferred face-to-face learning (47.8%; Table S1).

Private and public university lecturers in the lecturer group believed online learning is more modern and time-saving. In contrast, they thought face-to-face learning is more fun and interactive, has better knowledge transfer and students' attention, and has fewer technical issues. Private lecturers also thought face-to-face learning was more efficient and less tense and stressful, but public lecturers believed that online and face-to-face learning are similar in terms of work stress (Table S2).

#### 3.1.7. Preferred Amount of Online Learning in the Future Curriculum (Independent of COVID-19)

Dental undergraduates and lecturers from private and public universities agreed that all theoretical aspects of education, as well as case discussions or seminars, can be conducted online in the future, independent of the pandemic. Demonstrations for practicals, on the other hand, would be better done face-to-face. Furthermore, they believed each online teaching session should be limited to less than 2 h and no more than five lessons daily (Table 5). There was a significant difference between the private and public dental undergraduates' perception of the optimal amount of online learning in the future, independent of COVID-19 observed (*p*  < 0.05), with private university students showing a more positive perception (Table 6). There was no difference in lecturers' perceptions toward the same (*p*  > 0.05; Table 7).

### 3.2. Qualitative Phase

A total of four themes were extracted from the transcribed data after qualitative analysis. The themes could be broadly categorized into advantages (flexibility and student-centered learning) and limitations (inadequacy and lack of student engagement/interaction).

#### 3.2.1. Advantages

##### 3.2.1.1. Flexibility

One-third of undergraduates from private universities preferred online learning over face-to-face learning, while only one undergraduate from a public university preferred online learning. However, both the private and public undergraduates agreed that online learning saves travel time and provides them with more free time which they elaborated upon in the following statements:

*“I lived quite far from the campus. I have to take the train and travel for an hour to reach the campus, and you know that the train will be extremely crowded, especially during the working hours at 8 am. Through online learning it saves my time.”*

*“To add on, online learning is more comfortable as it can be done anytime, anywhere, in their preferred conducive environment.”*

*“I agree with the statement, I personally preferred to study alone in a relaxing state without any distractions, I can as well utilise my time according to my needs.”*

On the other hand, two out of six lecturers from private universities showed interest in online learning over face-to-face learning. These are the ideas suggested in the following:

*“For me as a lecturer and a mother, I always have to take care of both my career and children and through online learning it really makes my time more flexible and more cost saving from travelling.”*

*“In my opinion, I am currently working on a few research projects, time is always not enough for me, online learning is truly a good alternative as it really saves my time.”*

In contrast, none of the lecturers from public universities liked online learning. One public lecturer suggested the following:

*“As of my point of view, online learning is more beneficial and convenient for small-group discussions only. It is true that online learning is time-saving and saves travel costs, but in a group of 60 students, it is hard to ensure that all of them are focusing, and we didn't know what they were doing during the class.”*

##### 3.2.1.2. Student-Centered Learning

All the undergraduates from private and public universities agree that online learning allows asynchronous learning. They revealed the following:

*“Let's say if we missed something in the lecture, we can always have the time to watch back, and we can also watch at a faster speed.”*

*“For recorded online preclinical skills lab demonstrations, it allows us to revise before going for hands-on activities and competency tests.”*

Meanwhile, the lecturers from private and public universities believed that online learning promotes self-directed learning in students. They expressed the following:

*“Students can become more proactive in learning since they get to search online when they face difficulties immediately.”*

*“Self-directed learning can be achieved since the online lectures were being recorded; hence, students could always refer back to asynchronous studying materials (i-lectures and recorded demo sessions), revise by themselves, and ask us questions via emails or WhatsApp to solve their doubts.”*

#### 3.2.2. Limitations

##### 3.2.2.1. Inadequacy

Five out of six undergraduates from public universities have brought up the idea that online learning cannot cover certain aspects of skill-based education, and the same goes for private university students. A few participants shared their opinions regarding this matter:

*“In my opinion, I noted that the learning experience and the environment we undergo in a dental stimulatory lab setup differs from virtual learning; hence, I preferred physical, practical demonstrations, not online ones.”*

*“I agreed; I preferred face-to-face demos since we can appreciate the angulation of bur during tooth preparation and the proper setup of the working station directly.”*

Besides, online learning is resource-intensive, requiring more advanced equipment and a stable internet connection for a more precise projection during online demos. One proposed the following:

*“In this modern era, I'm sure that all of us will at least have an electronic gadget, but online learning needs a stable internet connection. Sometimes, when the line is not good enough, the projected screen will either be delayed or blurred, or the lecturer's voice will break. These affect the learning process.”*

All the lecturers were of one mind in which online learning failed to cover some aspects of skill-based education. They expressed their views as follows:

*“I was more keen on face-to-face learning regarding hands-on activities because through online learning, we cannot assess students' understanding as we can't appreciate the students' facial expressions, particularly eye contact.”*

*“Furthermore, the students need to have a 3D picture of the specific procedure and a direct experience of the learning environment in the lab and clinic in person.”*

*“For me, I think that online learning is much inferior for students to develop communication skills as they cannot appreciate body language and show empathy during online simulated sessions.”*

##### 3.2.2.2. Student Engagement/Interaction

The undergraduates agreed on the lack of engagement and interaction during online learning. One public university undergraduate shared the following statement:

*“A few students will always dominate the session and the others will remain silent, possibly due to a lack of confidence in their answers.”*

One of the private dental undergraduates also mentioned that:

*“Students who were shy in asking questions might leave it uncleared and affect their studies.”*

All personal dental undergraduates suggested that limited attention span is a common problem faced during online learning, while only two undergraduates from public universities think so. The following statement elaborated upon this:

*“I felt that online learning has more distraction as we might be multitasking, and the longer the duration of the particular session is, the easier it is for us to lose focus.”*

*“The student's attendance was also relatively lower than in face-to-face learning, particularly 8 a.m. sessions.”*

From the lecturers' point of view, all lecturers agreed on the lack of student engagement and interaction during online learning. The following statements support the above idea:

*“This could probably be due to shyness in the students speaking up and lack of self-esteem.”*

Two lecturers from public universities suggested that:

*“A lack of student response happened more frequently in big class groups than in small group learning, for instance, during problem-based learning (PBL).”*

*“Students always have a limited attention span during online learning when the online classes are scheduled for too long due to online fatigue.”*

*“Their attention span might be related to the our way of delivering the learning materials and the topic of discussion.”*

In the scenario where turning on the camera during online learning is mandatory, private and public university lecturers stood for different opinions. All public lecturers thought it was compulsory to switch on the camera to solve the problem of limited attention; however, they did mention that it does not apply to extensive group discussions. On the other hand, only two lecturers from private universities agreed with the statement, and the others stated that turning on the camera is totally up to students' preference.

## 4. Discussion

The worldwide spread of the recent pandemic has resulted in the unprecedented closure of academic institutions. Teaching and learning processes were transformed into virtual modalities to prevent overcrowding and reduce the risk of spreading infection. The students and faculty members opted to adapt to the new norm [14, 25]. Most previous studies solely focused on the student's perspective, and only a few looked at students' and lecturers' perspectives [26–29]. However, we documented and compared perceptions toward online learning among private and public university undergraduates and lecturers. The private and public university undergraduates were consistent in their attitude toward online learning regarding better flexibility. Further, both groups of students were agreeable regarding the time saved, which can help them to plan and study more. These findings are consistent with earlier research, which has shown that online learning allows greater flexibility in the place of the study process, time, and cost savings as it eliminates the need to travel to and from the campus [13].

However, the perceptions toward handling domain and motivation domain students were more positive in public university students than private university students. This could be because private universities rely on student tuition fees, as opposed to public colleges, which depend virtually entirely on government financial transfers. As students and families invest more in private universities, students' perspectives may differ [30], and they might not feel comfortable with online education. During the implementation of online learning, most students and lecturers believed that psychomotor skills could not be upgraded through online education, and face-to-face sessions were preferred.

An interesting point to note was that students and lecturers reported low self-efficacy during online lectures. Students' attention span and quality of online learning are strongly associated with the lecturers' ways of delivering the learning materials and the attractiveness of their prepared topic for discussion. Besides, peer-to-peer interaction and engagement in a group discussion were challenging to implement because of the intensive nature of the curriculum and the corresponding student learning time in most curriculums. Distraction, decreased interaction, communication, attendance, and shorter focus spans were among the factors that could make online learning less successful.

Independent of the pandemic scenario, private and public undergraduates have jointly opined that each online learning session should not be more than an hour. Two-hour lectures can be divided into two sessions with short breaks, as the level of attention during online learning will drop rapidly. Students' attention span has always been a matter of debate, as several experts have opined that 15 min is the maximum one can hold listeners' attention [31]. The lecturers suggested online synchronous lectures should be limited to a maximum of 20% of the curriculum; however, asynchronous recorded online courses can be beneficial, especially for preclinical skilled practical demonstrations, as they offer students a chance to revise before face-to-face hands-on skill training sessions.

The results of this study revealed crucial findings that can help to improve and develop learning strategies in the future. However, the current study had a few limitations. To begin with, the study's generalizability was hampered by the use of data from a single university, respectively, from private and public sectors. Second, although undergraduates and lecturers were encouraged to participate in this study, it was voluntary. Hence, the 32.4% response rate fell short of the planned goal of at least 50%. As a result, the number of nonrespondents may have compromised the study's power, and potential response bias cannot be ruled out [32]. Third, the Cronbach alpha for the lecturer questionnaire was below 0.7 (0.678). This might be because we did not get enough lecturers to participate in the study, as participation was entirely voluntary. Our estimated sample size was 44, whereas we only had 33 lecturers willing to participate in the study. Therefore, the study's findings have to be evaluated with caution. Further, the study's questionnaire only examined undergraduates' perceptions. It was unclear how distant learning affected undergraduates' academic performance and whether undergraduates faced difficulties understanding course learning objectives. Students' perceptions of course learning objectives sometimes differ from their comprehension. With that being the case, evaluating the effect of online learning on student academic achievement is just as important as assessing curricular transformation. Despite these constraints, the findings of this study provide valuable information on dental undergraduates' and lecturers' current perceptions of online learning methods used during the pandemic. Acceptance of learning methods by students and lecturers is crucial in developing a productive learning environment [33, 34]. Since infectious diseases continually emerge, academic institutions need to have an appropriate plan in place based on the student's and lecturers' expectations and ease.

## 5. Conclusion

The study showed that despite certain challenges, the undergraduates and lecturers could adjust to the online learning environment and mainly reflected a positive perspective on implementing online learning regardless of the university background. Though there were mild differences in the perceptions of dental undergraduates between private and public universities, lecturers' perceptions showed no difference. Confinement was a novel encounter for them, and the present online learning was a distinct pedagogical experience. Undergraduates and lecturers enjoyed the new norm to some extent. However, students still believed online learning is not an alternative to face-to-face preclinical and clinical practice as they missed numerous educational experiences. However, combining physical and online classes in terms of blended learning courses might be the future trend for dental education.

## Figures and Tables

**Table 1:** Domains and descriptive statistics of undergraduates' questionnaire.

Domain	Domain's description	M	SD	*N*
Handling	H1: The technical introduction (“tech checks”) in the first week of the semester prepared me well for online learning.	3.42	0.994	227
	H2: The software requirements for online learning were economically achievable.	3.94	0.799	227
	H3: I was able to prepare myself well in advance for the online learning (by script or book).	3.47	0.894	227
	H4: The online learning was structured well.	3.57	0.830	227
	H5: The aspiration level of online learning was good.	3.38	0.915	227
	H6: The image and sound quality of online learning was good.	3.58	0.871	227

Didactic benefit	D1: In the current situation, online learning was a good option for learning the theoretical part of education.	3.89	0.924	227
	D2: By participating in online learning, I feel well prepared for the practical part of education.	2.94	1.009	227
	D3: In the context of online learning I dare to ask questions more often than “face-to-face.”	3.12	1.168	227
	D4: I generally prefer “face-to-face” rather than online learning.	3.72	1.124	227
	D5: I do not think that online learning is useful and would have preferred a “non-semester” (extended sem) and (if possible) continuing with normal face-to-face learning.	2.77	1.179	227
	D6: I find online learning is more distracting than “face-to-face” teaching.	3.70	1.151	227
	D7: Online learning is more interactive than “face-to-face.”	2.63	1.037	227
	D8: Online learning expanded my knowledge well beyond “face-to-face.”	2.90	0.912	227
	D9: I did not encounter any difficulties in adapting online learning since the pandemic.	2.81	1.138	227
	D10: I think that online learning is more useful due to the policy of recording each session for future references, which aids in clearing my doubts without asking the lecturers.	4.04	0.954	227
	D11: I prefer online teaching because of time (traveling time).	3.80	1.049	227
	D12: I managed to attend all online classes despite no attendance being taken.	3.59	1.188	227
	D13: I perceived the knowledge and technique of doing lab projects better through video demonstration than through face-to-face.	3.00	1.175	227
	D14: During online teaching, I can immediately Google Search when I face difficulties in certain topics to clear my doubt so that I can understand that topic clearer.	4.10	0.800	227

Motivation	M1: The use of new digital teaching methods (e.g., online teaching) motivates me to learn.	3.40	0.913	227

Abbreviations: M, mean; *N*, number of valid answers (total: *N* = 227); SD, standard deviation.

**Table 2:** Domains and descriptive statistics of lecturers' questionnaire.

Domain	Domain's description	M	SD	*N*
Handling	H1: The technical introduction (“tech checks”) in the first week of the semester prepared me well for online learning.	3.73	0.876	33
	H2: The software requirements for online learning were economically achievable.	4.12	0.857	33
	H3: I was able to prepare myself well in advance for the online learning (by script or book).	3.85	0.795	33
	H4: The online learning was structured well.	3.85	0.619	33
	H5: The aspiration level of online learning was good.	3.58	0.792	33
	H6: The image and sound quality of online learning was good.	3.76	0.614	33

Didactic benefit	D1: In the current situation, online learning was a good way to teach the theoretical part of education.	4.42	0.561	33
	D2: The theoretical teaching content could be covered just as well by online teaching formats as it would have been possible in a classroom course (lecture/seminar).	3.91	1.071	33
	D3: I found the students during the online teaching to be disciplined and attentive.	2.79	0.960	33
	D4: I feel more uncomfortable using new teaching methods such as online teaching than in “face-to-face” teaching such as lectures because I miss direct communication with the students.	3.33	1.339	33
	D5: I do not think that online learning is useful and would have preferred a “non-semester” and (if possible) the continuation of “normal teaching.”	2.33	1.051	33
	D6: I find that online teaching is more challenging than delivering “face-to-face.”	3.39	1.171	33
	D7: Response rate from students during online learning is much more disappointing than “face-to-face.”	3.64	1.168	33
	D8: Attendance rate from students during online learning is much more disappointing than “face-to-face.”	3.12	1.023	33
	D9: The flow of online teaching is comparable as smooth as delivering “face-to-face.” (technical issues)	3.27	0.911	33
	D10: I prefer online teaching because of time-saving (traveling time).	3.73	1.281	33
	D11: More interactions with the students because they tend to ask more questions via online learning compared to face-to-face.	2.97	1.262	33
	D12: Explanation can be more detailed as I can screen share my device and search for related pictures and photos of certain topics.	3.91	0.980	33

Motivation	M1: The use of new digital teaching methods (e.g., online teaching) motivates me.	3.88	0.781	33
	M2: The online learning formats are an added value because I can also use them for other training purposes.	4.15	0.619	33

Abbreviations: M, mean; *N*, number of valid answers (total: *N* = 33); SD, standard deviation.

**Table 3:** Comparison of domains among dental undergraduates between private and public university.

Domains	University	Mean rank	*p*-Value
Handling	Private	98.90	**0.005**
	Public	123.74	

Didactic benefit	Private	113.67	0.952
	Public	114.21	

Motivation	Private	101.30	**0.013**
	Public	122.19	

Total	Private	106.31	0.156
	Public	118.96	

*Note*: Bold values signify *p* < 0.05.

**Table 4:** Comparison of domains among lecturers between private and public university.

Domains	University	Mean Rank	*p*-Value
Handling	Private	15.15	0.254
	Public	18.97	

Didactic benefit	Private	15.71	0.426
	Public	18.38	

Motivation	Private	15.47	0.326
	Public	18.63	

Total	Private	15.44	0.338
	Public	18.66	

**Table 5:** Descriptive statistic of preferred amount of online learning in the future curriculum (independent of COVID-19).

Undergraduates' perspective			Statements	Lecturers' perspective		
M	SD	*N*		M	SD	*N*
3.35	1.143	227	All theoretical parts of education will be carried out online.	3.52	1.149	33

3.50	1.176	227	Case discussions or seminars will be carried out online.	3.85	1.093	33

2.72	1.386	227	Demos for practicals will be carried out online.	2.76	1.415	33

4.31	0.873	227	The duration of each online teaching session is limited to less than 2 h.	4.52	0.667	33

4.29	0.933	227	Daily maximum number of online classes is limited to less than five classes.	4.12	0.960	33

Abbreviations: M, mean; *N*, number of valid answers (Total: Undergraduates *N* = 227, Lecturers *N* = 33); SD, standard deviation.

**Table 6:** Comparison of dental undergraduates' perception on the preferred amount of online learning in the future curriculum independent of COVID-19 between private and public university.

Statements	University	Mean	*p*-Value
All theoretical parts of education will be carried out online.	Private	127.44	0.011
	Public	105.33	

Case discussions or seminars will be carried out online.	Private	128.89	**0.005**
	Public	104.39	

Demos for practicals will be carried out online.	Private	133.65	**<0.001**
	Public	101.33	

Duration of each online teaching session limited to less than 2 h.	Private	129.94	**0.001**
	Public	103.72	

Daily maximum no. of online classes limited to less than five classes.	Private	135.24	**<0.001**
	Public	100.30	

*Note*: Bold values signify *p* < 0.05.

**Table 7:** Comparison of dental lecturers' perception on the preferred amount of online learning in the future curriculum independent of COVID-19 between private and public university.

Statements	University	Mean rank	*p*-Value
All theoretical parts of education will be carried out online.	Private	15.59	0.365
	Public	18.50	

Case discussion or seminars will be carried out online.	Private	16.79	0.892
	Public	17.22	

Demos for practicals will be carried out online.	Private	18.62	0.310
	Public	15.28	

Duration of each online teaching session limited to less than 2 h.	Private	15.68	0.349
	Public	18.41	

Daily maximum no. of online classes limited to less than five classes.	Private	17.35	0.818
	Public	16.63	

## Data Availability

Data are available on reasonable request from the corresponding author.

## Supporting Information

Supporting Information Table S1: The percentage of dental undergraduates' overall assessment. Table S2: The percentage of dental lecturers' overall assessment.
