# Supplementary material for: Online Learning in Dental Education: Comparison of Perspectives of Lecturers and Undergraduate Students Between a Public and Private University: A Mixed-Method Study
Source: Int J Dent. 2024 Oct 16;2024:7389743. doi: 10.1155/2024/7389743 (PMC11498980; doi:10.1155/2024/7389743)
Supplement: Supporting Information — Table S1: The percentage of dental undergraduates' overall assessment. Table S2: The percentage of dental lecturers' overall assessment. [file 7389743.f1.docx]

***Supplementary table 1:*** *The percentage of* ***dental undergraduates’*** *overall assessment*

| **Variable** | **Overall assessment** | **Private university(%)** | **Public university(%)** | **Total(%)** |
| --- | --- | --- | --- | --- |
| **More modern** | Online learning | 56.2 | 52.9 | 54.2 |
|  | Equivalent | 30.3 | 28.3 | 29.1 |
|  | Face-to-face | 13.5 | 18.8 | 16.7 |
| **More fun** | Online learning | 9.0 | 8.7 | 8.8 |
|  | Equivalent | 44.9 | 32.6 | 37.4 |
|  | Face-to-face | 46.1 | 58.7 | 53.7 |
| **More tips of lecturers** | Online learning | 19.1 | 7.2 | 11.9 |
|  | Equivalent | 55.1 | 36.2 | 43.6 |
|  | Face-to-face | 25.8 | 56.5 | 44.5 |
| **Queries better possible** | Online learning | 25.8 | 15.2 | 19.4 |
|  | Equivalent | 47.2 | 52.2 | 50.2 |
|  | Face-to-face | 27.0 | 32.6 | 30.4 |
| **Better knowledge transfer** | Online learning | 19.1 | 13.8 | 15.9 |
|  | Equivalent | 47.2 | 31.9 | 37.9 |
|  | Face-to-face | 33.7 | 54.3 | 46.3 |
| **Easier participation** | Online learning | 34.8 | 34.8 | 34.8 |
|  | Equivalent | 36.0 | 26.1 | 30.0 |
|  | Face-to-face | 29.2 | 39.1 | 35.2 |
| **Less time effort** | Online learning | 67.4 | 55.8 | 60.4 |
|  | Equivalent | 14.6 | 24.6 | 20.7 |
|  | Face-to-face | 18.0 | 19.6 | 18.9 |
| **More interactive** | Online learning | 9.0 | 9.4 | 9.3 |
|  | Equivalent | 42.7 | 31.9 | 36.1 |
|  | Face-to-face | 48.3 | 58.7 | 54.6 |
| **Better focusing** | Online learning | 19.1 | 17.4 | 18.1 |
|  | Equivalent | 31.5 | 24.6 | 27.3 |
|  | Face-to-face | 49.4 | 58.0 | 54.6 |
| **Less tension and stress** | Online learning | 44.9 | 26.8 | 33.9 |
|  | Equivalent | 40.4 | 44.2 | 42.7 |
|  | Face-to-face | 14.6 | 29.0 | 23.3 |
| **More efficient** | Online learning | 43.8 | 15.9 | 26.9 |
|  | Equivalent | 28.1 | 36.2 | 33.0 |
|  | Face-to-face | 28.1 | 47.8 | 40.1 |
| **Less technical issues** | Online learning | 16.9 | 13.8 | 15.0 |
|  | Equivalent | 20.2 | 16.7 | 18.1 |
|  | Face-to-face | 62.9 | 69.6 | 67.0 |

***Supplementary table 2:*** *The percentage of* ***dental lecturers’*** *overall assessment*

| **Variable** | **Overall assessment** | **Private university(%)** | **Public university(%)** | **Total(%)** |
| --- | --- | --- | --- | --- |
| **More modern** | Online learning | 64.7 | 62.5 | 63.6 |
|  | Equivalent | 11.8 | 12.5 | 12.1 |
|  | Face-to-face | 23.5 | 25.0 | 24.2 |
| **More fun** | Online learning | 23.5 | 18.8 | 21.2 |
|  | Equivalent | 29.4 | 37.5 | 33.3 |
|  | Face-to-face | 47.1 | 43.8 | 45.5 |
| **More tips of lecturers** | Online learning | 17.6 | 6.3 | 12.1 |
|  | Equivalent | 41.2 | 56.3 | 48.5 |
|  | Face-to-face | 41.2 | 37.5 | 39.4 |
| **Queries better possible** | Online learning | 23.5 | 6.3 | 15.2 |
|  | Equivalent | 41.2 | 56.3 | 48.5 |
|  | Face-to-face | 35.3 | 37.5 | 36.4 |
| **Better knowledge transfer** | Online learning | 11.8 | 6.3 | 9.1 |
|  | Equivalent | 35.3 | 43.8 | 39.4 |
|  | Face-to-face | 52.9 | 50.0 | 51.5 |
| **Easier participation** | Online learning | 41.2 | 31.3 | 36.4 |
|  | Equivalent | 23.5 | 25.0 | 24.2 |
|  | Face-to-face | 35.3 | 43.8 | 39.4 |
| **Less time effort** | Online learning | 41.2 | 50.0 | 45.5 |
|  | Equivalent | 35.3 | 18.8 | 27.3 |
|  | Face-to-face | 23.5 | 31.3 | 27.3 |
| **More interactive** | Online learning | 23.5 | 12.5 | 18.2 |
|  | Equivalent | 17.6 | 43.8 | 30.3 |
|  | Face-to-face | 58.8 | 43.8 | 51.5 |
| **Better focusing** | Online learning | 5.9 | 12.5 | 9.1 |
|  | Equivalent | 29.4 | 37.5 | 33.3 |
|  | Face-to-face | 64.7 | 50.0 | 57.6 |
| **Less tension and stress** | Online learning | 29.4 | 18.8 | 24.2 |
|  | Equivalent | 29.4 | 56.3 | 42.4 |
|  | Face-to-face | 41.2 | 25.0 | 33.3 |
| **More efficient** | Online learning | 23.5 | 12.5 | 18.2 |
|  | Equivalent | 29.4 | 56.3 | 42.4 |
|  | Face-to-face | 47.1 | 31.3 | 39.4 |
| **Less technical issues** | Online learning | 0 | 0 | 0 |
|  | Equivalent | 29.4 | 18.8 | 24.2 |
|  | Face-to-face | 70.6 | 81.3 | 75.8 |
